# The N-terminal BRCT domain determines MCPH1 function in brain development and fertility

**DOI:** 10.1038/s41419-021-03406-3

**Published:** 2021-02-01

**Authors:** Xiaoqian Liu, Nadine Schneble-Löhnert, Martina Kristofova, Xiaobing Qing, Jan Labisch, Susanne Hofmann, Sandra Ehrenberg, Mara Sannai, Tjard Jörß, Alessandro Ori, Maren Godmann, Zhao-Qi Wang

**Affiliations:** 1 Leibniz Institute on Aging - Fritz Lipmann Institute (FLI), Beutenbergstrasse 11, 07745 Jena, Germany; 2 Institute of Biochemistry and Biophysics, Department of Biochemistry, Friedrich-Schiller-University of Jena, Hans-Knöll-Str. 2, 07745 Jena, Germany; 3 Faculty of Biological Sciences, Friedrich-Schiller University of Jena, Beutenbergstrasse 11, 07745 Jena, Germany

**Keywords:** Disease model, Genetics of the nervous system

## Abstract

MCPH1 is a causal gene for the neurodevelopmental disorder, human primary microcephaly (MCPH1, OMIM251200). Most pathogenic mutations are located in the N-terminal region of the gene, which encodes a BRCT domain, suggesting an important function of this domain in brain size determination. To investigate the specific function of the N-terminal BRCT domain in vivo, we generated a mouse model lacking the N’-BRCT domain of MCPH1 (referred as *Mcph1*-ΔBR1). These mutant mice are viable, but exhibit reduced brain size, with a thinner cortex due to a reduction of neuroprogenitor populations and premature neurogenic differentiation. *Mcph1*-ΔBR1 mice (both male and female) are infertile; however, almost all female mutants develop ovary tumours. *Mcph1*-ΔBR1 MEF cells exhibit a defect in DNA damage response and DNA repair, and show the premature chromosome condensation (PCC) phenotype, a hallmark of MCPH1 patient cells and also Mcph1 knockout cells. In comparison with *Mcph1* complete knockout mice, *Mcph1*-ΔBR1 mice faithfully reproduce all phenotypes, indicating an essential role of the N-terminal BRCT domain for the physiological function of MCPH1 in the control of brain size and gonad development as well as in multiple cellular processes.

## Introduction

Human primary microcephaly (MCPH, OMIM251200) is a neurodevelopmental disorder characterised by a marked reduction of brain size, especially reduced size of the cerebral cortex, yet with normal brain architecture and non-progressive intellectual disability. MCPH has a 1:30,000 to 1:250,000 incidence per live-birth, depending on the population^1–3^. So far, 25 gene loci have been identified to be potentially involved in the occurrence of MCPH^4–7^.

The *MCPH1/BRIT1* gene is the first gene reported to be responsible for MCPH type 1 (MCPH1, OMIM251200) and also for another syndrome called premature chromosome condensation syndrome (PCC, OMIM 606858), because MCPH1 patient cells exhibit prophase-like and premature chromosome condensation^1,8–11^. The human *MCPH1* gene, located in human chromosome 8p23.1, contains 14 exons and encodes 835aa (amino acid) of the full length MCPH1 protein. MCPH1 contains several functional domains; the mid region binds to condensin II, TopBP1, Chk1 and βTrCP2^12,13^ and three BRCA1 C-terminus (BRCT) domains—an evolutionarily conserved phospho-peptide interacting domain—which are frequently found in proteins involved in DNA damage response (DDR) and cell cycle control^1,14,15^. The N-terminal BRCT domain (13aa to 89aa) is implicated in DNA repair via interaction with chromatin remodelling complex SWI/SNF^16^ and allows MCPH1 to localise to centrosomes in response to DNA damage^17^. The two C-terminal tandem BRCT domains encoded by exons12–14, ranging from 672aa to 730aa and from 751aa to 833aa respectively^1,15,18,19^, are necessary for oligomer formation and ionising radiation (IR)-induced foci (IRIF) by interacting with γH2AX during the DDR and also in E2F1-mediated transcriptional regulation of Chk1 and BRCA1 in G2/M checkpoint^14,18,20,21^.

MCPH1 has been shown to play an important role in brain development, gonad formation and tumorigenesis in transgenic mouse models. Mice carrying a deletion of exon 2 (designated hereafter as *Mcph1*-ko)^22^ showed no obvious microcephaly phenotype, but exhibited growth retardation and infertility. Mutant male mice had atrophic testes, characterised by thinner testicular tubules and absence of spermatocytes, due to impaired DNA repair that leads to failure of chromosomal synapsis, meiosis arrest and apoptosis. Moreover, *Mcph1*-ko female ovaries were small and often lacked ovarian follicles. About 17% of *Mcph1*-ko mice developed malignant tumours, originated from lymphomas and ovary tumours over a period of 2.5 years^22^. We generated *Mcph1* null mice (designated hereafter *Mcph1*-Δ)^23^ by deleting exons 4 and 5. These mice were viable and showed a microcephaly phenotype associated with a thinner neocortex and premature differentiation of neuroprogenitors, due to MCPH1-TrCP2-Cdc25 mediated premature mitosis entry and the switch of division mode from symmetric to asymmetric^12,23^. Like human MCPH1 cells, which are defective in the DNA damage response^20,24,25^, *Mcph1*-Δ neuroprogenitors are sensitive to irradiation-induced DNA damage, which enhanced apoptosis in *Mcph1*-Δ cortex^26^; *Mcph1*-Δ cells showed a classic PCC phenotype. Moreover, *Mcph1*-Δ mice were infertile and strikingly exhibited a very high incidence (94.4%) of ovary tumours (granulosa cell tumours and Sertoli-Leydig cell tumour) in female mice over a period of 18 months^27^. Another mouse model called *Mcph1*^tm1a^, generated by deleting exon 4 of the *Mcph1* gene, exhibited less brain weight and a moderate defect of hearing loss^28^. It is worth mentioning that a mouse model lacking the C-terminal BRCT domain (*Mcph1*^gt/gt^ mice) showed a reduced survival rate, without affecting the brain size^29^, suggesting that the C-terminal BRCT domain is not important for brain development. Nevertheless, *Mcph1*^gt/gt^ cells showed a PCC phenotype—a characteristic of MCPH1^29^—indicating that this C’-BRCT is responsible for PCC, which can separate MCPH1 from its function in brain development. Combined with findings in human MCPH1 cells, these mouse model studies indicate that MCPH1 is a multi-faceted protein and different regions may control the specific function of MCPH1, ranging from brain development, cell cycle progression, fertility and tumorigenesis.

A total of 12 mutations were identified in MCPH1 patients, with all located in exons 1 to 6. Seven of these mutations caused code frameshift or loss of start transcription site leading to loss of MCPH1 proteins; whereas 5 missense mutations leading to a single amino acid substitution at the N-terminal BRCT domain of MCPH1, were found in human MCPH1 patients^1,10,13,27,30–33^. The mutation spectrum in the N-terminal domain of the MCPH1 protein, strongly suggests an important function of N-terminal BRCT domain in preventing the microcephaly phenotype.

This study investigated the function of the N-terminal BRCT domain by specifically deleting it in the mouse genome (*Mcph1*-ΔBR1 mice). These mutant mice developed primary microcephaly, accompanied with a thinner neocortex, a reduction of the neuroprogenitor pool and premature neuronal differentiation during brain development. *Mcph1*-ΔBR1 mice showed a block in reproductive organ development, characterised by testis atrophy and lacking ovaries. Intriguingly, almost all mutant females developed ovary tumours. At the cellular level, *Mcph1*-ΔBR1 MEF (mouse embryonic fibroblast) cells exhibited the PCC phenotype and defects in cell proliferation and the DDR. All these phenotypes identify the N-terminal BRCT domain as a major regulator of brain size, gonad development and tumour repression.

## Materials and methods

### Mice

Mice were maintained in the mouse facility of Fritz Lipmann-Institute (FLI, Jena, Germany) and fed *ad libitum* with standard laboratory chow and water in ventilated cages under a 12 hr light/dark cycle. All animal experiments and breeding were conducted according to the German animal welfare legislation and approved by the Thüringer Landesverwaltungsamt. Genotype analysis for *Mcph1*-Δ mice were determined by PCR on DNA extracted from tail tissue, as previously described^23^.

### Gene targeting in ES cells and generation of *Mcph1*-ΔBR1 mice

For construction of a mouse model carrying a deletion of the N-terminal BRCT domain of MCPH1, the targeted vector was constructed with 5′ homologous arm (5HR) containing exon1 and 3HR containing the entire sequence from exon 4 to exon 6. The targeted mice were obtained through electroporation of this vector into embryonic stem cells, screening for corrected targeted ES clones and injection of targeted ES clones into blastocysts as previously described^23^. The knocked-in allele was obtained by crossing with Stra8-Cre mice to delete the Neo cassette. Homozygous Ki/Ki mice were generated by breeding between + /Ki mice. All animal experiments were conducted according to the German animal welfare legislation.

### Genotyping and cDNA sequencing of *Mcph1*-ΔBR1 mice by PCR

Genome DNA was extracted from mouse tails as previously described^23^. PCR was performed to determine the genotype of the *Mcph1*-ΔBR1 mice. Primer combinations amplified three distinct bands: WT = 312 bp, Tg = 582 bp and Ki = 426 bp. The primers were as follows: Fwd Ex1: 5′- GTGTGGACACCCCACATACA−3′; I1 as: 5′- AGGGTCCCATGTTCTGAGC−3′; Rev NEO: 5′- TCGTCCTGCAGTTCATTCAG−3′; Rev Ex4: 5′-ATCGGTATTCACTGCAGGGAA−3′. Primers for internal control were: Ctr-F: 5’- CTAGGCCACAGAATTGAAAGATCT−3′; Ctr-R: 5′- GTAGGTGGAAATTCTAGCATCATCC−3′. Primers for the Cre transgene were: Stra-F: 5′- GTGCAAGCTGAACAACAGGA−3′; Stra-R: 5′- AGGGACACAGCATTGGAGTC−3′. Primer for cDNA sequencing: mMCPH1–1F: CCCTCGAGATGGAGGCCTCGGGAGGCGTTG, mMCPH1–8R: CCCTCGAGTTTCACTATATCCTGTGGC.

### Southern blotting

Southern blot was performed as previously described^23^. Briefly, 10 μg of genome DNA was digested with 30 units of the corresponding enzymes at 37 °C overnight. The samples were electrophoresed in 0.8% agarose gel. After denaturation and depurination, the gel was transferred to Amersham Hybond membrane XL (#RPN2020S, GE healthcare life science), which then was hybridised with indicated probes after labelling with α-^32^P dCTP (Hartmann Analytic) using Prime-a-Gene® Labelling System (#U1100, Promega), as per manufacturer’s protocol and the Amersham NICK Columns (108–0855–02, GE healthcare life science). The blot was visualised by exposure to FUJIFLIM or by Phospho-imaging machine (FUJI).

### Probes for Southern blotting

Probes were designed by using software and NCBI Blast online, amplified by PCR using both forward and reverse primers, cloned to pGEM®-T Easy Vector (Promega) and sequenced. The probe vectors were digested by EcoRI and purified for Southern blotting.

5P4 was an external probe which is located at 5′-prime upstream of the targeted region (intron 1). The sequence of 5P4 was as follows:

TTGGTGCTGGCCTCTAAATCTGACGTTCAGGAGCTTCTGGAAACCCAGGTCTAGGCCCCGCTGTCTTCAGTGTCTGGGCCACACTGTTGAGAGTGCAGGTTTCTGGGGGAACTTCAGACTGTGGGGAGCCAGTGTTCCCCCTCTGATGCAGCGGACCCTTTAGACCCTTTTTATCTGACTTGTTTGCCAGGACTTCTTGGAGACTCGACTGCTGCTGATTCCTGCCTGCTGTCCAAATGCTGTCAGGGTGTTAAGAAGAAATCTCTGAAAGCTAACCTCCCGCTGAGCCCACGTGTGCGCCTCACAAAAGTCAATATTTTCAGCTTTCCCTCGGCCCCAGTGTGTTTAAAGGGCTTTCTAGTGTACATCCTTTGATTGTGGGTGAGTGTTCATAAGGAAAATGGCTTCCATCTTTTGTCTGCAGAGAGTGAAAAAGAAGCAGAGTCGCTCTAGTTTCGTCTTCACCGGATTCTTTCTTTAGATGAAGTTA

3P4 was an external probe which is located at 3′-prime downstream of the targeted region (intron 7). The sequence of 3P4 was as follows:

ACACCCAGGCTTCTGACATAAGTAGTCTAAGAATTTATGCTCTTAGTGATGTGCCACACTGAATCGTGGCTTATTGAAGAGTGATTAATGCATTCAGTTCCTAAGAGTAAATCTCAGTATCACTCCAACTGCTGTCTTGGAAGCACTTCAGCAGAGGCCCAGTATCAGCTGACCTTTGCTTTTGCCCCTCCCTTCCCAGAGCACAGCTTCAAGGACAGGATATCTCTGTTGCCACCACTCTGCTCTGAAAGCCGAGCTCGAGTTGTTTAAGTTAGAATCCCAGCACTGCCTCCAGATGGTGTCTTTTCTCTAAAAAAGGAAGCCTTCAGCTGGGCAGCACTGTACCAGTAGAACTGGGTCTGAGTAATGACCACAGGTTCAGGAAAGTGAGCAGAAAGTGCTGACTGCCCTCTAGTACTTCAGTATAATGAGAGGACAGTAGCATGCTGAGTCCCAGGAGGGGCTAGCACTGTGACCTGACAGGATCAGGAGAGTCACAGGTGACACTGACACTGAGCTGGTTTTGAAGGATGAGTTGTGTTTACCCACAGCAAAATGTGTCAGAAAGCTGGGGTCAGAGTGGGGAGCCTGCAGAGGTGATGGACAGCGTATCATTACTTCTGAGAGAAGTGCAGACTTCATGAGAGATCGGAATATTGAGAGCCCCACATGCTTCATTTGAAATATAAATAAAACATGAGGACTTGATGGAAGAACAGGGGACAGATATGAGGAGCCGTGTTCTGTTT

### Preparation of peptides from E13.5 mouse cortex for mass spectrometry analysis

E13.5 cortex isolation was performed in PBS and the tissue lysed in lysis buffer (4% SDS (w/v), 100 mM HEPES, 50 mM DTT in milliQ water), then sonicated in a Bioruptor Plus (Diagenode) for 5 cycles of 60 sec ON and 30 sec OFF, followed by heating at 95 °C for 10 min. For reduction and alkylation of cysteines, lysates were incubated with 10 mM DTT at 45 °C for 15 min and incubated with 15 mM iodacetamide at room temperature for 30 min. Each sample was precipitated with ice-cold acetone (Biosolve #010306) overnight at −20 °C. Following centrifugation at 20,800 × *g* for 30 min at 4 °C, the pellets were washed twice with ice-cold 80% acetone and allowed to air-dry, before being dissolved in digestion buffer at 1 mg protein/ml using 1 M GuHCl in 100 mM HEPES, pH8. The suspension of the protein was digested for 4 hr at 37 °C using 1:100 (w/w) LysC (Wako Chemicals GmbH #125–05061). Samples were then diluted to 0.5 M GuHCl by milliQ water and digested with 1:100 (w/w) trypsin (Promega #V5111) for 16 hr at 37 °C. Digested peptide solutions were acidified with 10% TFA, then desalted in Waters Oasis® HLB μElution Plate (30 μm, Waters 186001828BA) under a slow vacuum. Before this process, the plate was pre-conditioned with 3 × 100 μl solvent B (80% acetonitrile from Biosolve #06914143) and equilibrated with 3 × 100 μl solvent A (0.05% formic acid in milliQ water). Samples were loaded, washed 3 times with 100 μl solvent A, and eluted into PCR tubes with 50 μl solvent B. Eluates were dried with a speed vacuum centrifuge and dissolved in 5% acetonitrile, 0.1% formic acid to a peptide concentration of 2 mg/ml.

### Parallel Reaction Monitoring (PRM) for MCPH1 peptides

To prepare standard peptides for PRM, proteotypic peptides for mouse MCPH1 were synthesised and isotopically labelled by incorporation of heavy Arginine (U-13C6;U-15N4) or Lysine (U-13C6; U-15N2) at the C-terminus (JPT Peptide Technologies GmbH, Berlin, Germany). Lyophilised peptides were reconstituted in 20% (v/v) acetonitrile, 0.1% (v/v) formic acid before being pooled together in equal amounts. An aliquot of the pooled peptides (~160 fmol per peptide on column) was analysed by shotgun liquid chromatography tandem mass spectrometry using both data-dependent and data-independent acquisition. The data were used for PRM assay development using SpectroDive v.9 (Biognosys AG) at default settings. PRM assays were successfully developed for 14 peptides derived from MCPH1 (see below).

**Table:** 

Protein Access	GeneID	Peptide sequence	Charge state
Q7TT79	Mcph1	AALDDDVPVLLFESPR	+2, +3
Q7TT79	Mcph1	ASSFYGSASPNHLR	+2, +3
Q7TT79	Mcph1	DATGAVADSER	+1, +2
Q7TT79	Mcph1	DGYQSTWDK	+1, +2
Q7TT79	Mcph1	ENIATGYSESVK	+2
Q7TT79	Mcph1	HLSTQQYQGTLFANQPK	+2, +3
Q7TT79	Mcph1	LPPEAQQLASPSLFHCR	+2, +3
Q7TT79	Mcph1	LVSVLWVEK	+1, +2
Q7TT79	Mcph1	QAAGVSQGVPDEK	+2
Q7TT79	Mcph1	QVTHVIFK	+2
Q7TT79	Mcph1	SALAIQLFK	+1, +2
Q7TT79	Mcph1	SDQSPPSTIR	+2
Q7TT79	Mcph1	SISSISDLISK	+2
Q7TT79	Mcph1	WVLDSITQHK	+2, +3

After assay development, the pool of heavy synthetic peptides was spiked into cortex derived peptides. iRT peptides (Biognosys AG) were also spiked into each sample prior to analysis by PRM-MS for retention time calibration. Peptides (1 μg) were separated using a nanoAcquity UPLC M-Class system (Waters) with a trapping (nanoAcquity Symmetry C18, 5 µm, 180 µm × 20 mm) and analytical column (nanoAcquity BEH C18, 1.7 µm, 75 µm × 250 mm). The outlet of the analytical column was coupled directly to a Q-exactive HF-X (Thermo Fisher) using the Proxeon nanospray source. Peptides were eluted via the analytical column with a constant flow of 0.3 μl/min. During this step, the percentage of acetonitrile (in 0.1% in formic acid) was increased in a non-linear fashion from 0% to 40% over 40 min. PRM acquisition was scheduled for the duration of the entire gradient using the “DIA” mode with the following settings: resolution 120,000 FWHM, AGC target 3 × 10^6^, maximum injection time (IT) 250 ms, isolation window 0.4 m/z. For each acquisition cycle, a “full MS” scan was acquired with the following settings: resolution 120,000 FWHM, AGC target 3 × 10^6^, maximum IT 10 ms, scan range 350 to 1650 m/z. Peak group identification and quantification were performed using SpectroDive v9. The summed height of all identified transitions was used to estimate the quantity of each peptide. Peptide quantities were normalised by dividing their integrated intensity with summed intensity of all samples of the Base Peak Chromatogram extracted for each sample from “full MS” scans, using Xcalibur v4.1. Only peptides reliably quantified across all replicates (identification q < 0.05 and at least 5 transitions identified) were retained for genotype comparison and protein quantification.

### Organ isolation and preparation for paraffin sections and cryosection

Adult mice were sacrificed by CO_2_ asphyxiation. Testes, ovaries and brains were isolated and fixed with 4% PFA at 4 °C overnight. The newborn brains (P1) were isolated by decapitation and the pregnant mice at E17.5 were sacrificed by cervical dislocation and embryonic brains isolated and fixed overnight with 4% PFA at 4 °C. The tissues were embedded in paraffin and sectioned. For preparation of cryosections, the fixed brains were transferred to a solution of 30% sucrose, embedded in a plastic mould using a cryomatrix (Neg −50, Richard-Allan Scientific) and sectioned using cryostate (Leica, Wetzlar, Germany). The cryosections were stored at −80 °C and processed for immunostaining.

### Immunohistochemistry

Hematoxylin and Eosin (H&E) staining of paraffin sections were performed as previously described^23^. For staining cryosections of mouse brain, the antigen was retrieved by using a 10 mM sodium citrate buffer (pH 6.0) and blocked in a blocking solution (5% goat serum, 1% BSA, 0.4% Triton X-100 in PBS). Cryosections were incubated with first antibodies Sox2 (1:200, #ab97959, Abcam), Tbr2 (1:200, #ab23345, Abcam) and Ctip2 (1:200, #ab18465, Abcam) at 4 °C overnight, followed by washing with PBS. Sections were then incubated with the secondary antibody anti-rabbit IgG’ fragment-Cy3 (1:200, C2306, Sigma-Aldrich) and anti-mouse IgG’ fragment-Cy2 (1:200, 711–225–152, Jackson)) for 2 hr at room temperature. The DNA was counterstained with DAPI (40,6-diamidino-2-phenylindole).

### Histological and immunochemical analyses of gonads

Testes and ovaries from control and mutant mice were harvested, fixed in Bouin’s solution, processed and paraffin embedded. Specimens were sectioned at 5 µm and H&E staining completed by the histology core facility of the FLI. Morphology and quantitative histological analyses were performed as described^34^. Briefly, only circular seminiferous tubules were included in the analysis and diameter of control and mutant tubular cross-sections measured using Nikon’s NIS-Elements imaging software. At least 30 tubular cross-sections per animal were analysed. To determine the number of damaged seminiferous tubules, vacuolised tubular cross-sections were counted and calculated as number per total seminiferous tubular cross-sections of a testis.

For immunostaining, samples were deparaffinized, rehydrated and microwaved in 0.01 M sodium citrate buffer, pH 6.0, for antigen retrieval. Endogenous peroxidase was inhibited with 0,03% H_2_O_2_/PBS. Unspecific antibody binding was blocked by incubation with 5% BSA/PBS or SEA block (ThermoFisher). Primary antibodies (anti-DDX4 (Abcam, ab13840, 1:800), anti-SOX9 (EMD Millipore, AB5535, 1:500), anti-3β-HSD (Santa Cruz Biotechnology, sc-30820, 1:200), anti-Ar (Santa Cruz Biotechnology, sc-816, 1:200), anti-CX43 (Cell Signaling Technology, 3512, 1:200), anti-LIN28 (Cell Signaling Technology, 3978, 1:400) were diluted in blocking buffer and incubated overnight at 4 °C. Control stainings were completed using IgG instead of the first antibody (mIgG (Santa Cruz Biotechnology, sc-2025), rbIgG (Santa Cruz Biotechnology, sc-2027)). After washing with PBS, samples were incubated with the secondary antibody (m/rb/g-HRP (Pierce) 1:1000; m-Alexa594, rb-Alexa488 (ThermoFisher) 1:500) for 1 h at room temperature. Antibody binding was visualised with a liquid DAB + Substrate Chromogen System (Dako). Nuclear counterstaining was performed with Mayer’s hemalum. Immunoassayed sections were analysed with Nikon Eclipse Ti microscope using Nikon’s NIS-Elements imaging software. Ddx4-positive germ cells were discriminated from Sox9-positive Sertoli cells. Spermatogonial stem cells were identified by Lin28 immunostaining. To determine the number of SSCs per seminiferous tubule, Lin28-positive SSCs and Sox9-positive Sertoli cells were counted in 30 circular cross-sections per animal, with results depicted as ratio (Lin28 + SSCs to Sox9+ Sertoli cells).

### Primary MEFs isolation and culture

Pregnant mice at E13.5 were sacrificed by cervical dislocation and the embryos removed and collected in a 6-well dish containing ice cold PBS. The foetal liver was collected for DNA isolation and PCR-genotyping to determine the genotype of the embryos. Embryos were transferred into a cryovial containing ice-cold PBS and chopped into small pieces. The chopped tissue was digested with trypsin-EDTA-solution (#25300, life technologies) for 4 min and the supernatant transferred to tubes containing MEF medium. This trypsinization procedure was repeated three more times and the cell supernatant collected in the same tube. Said cell supernatant was spun down and the cell pellets cultured in MEF medium (Dulbecco’s modified Eagle’s medium supplemented with 10% foetal bovine serum, 2 mM glutamine, 1 mM sodium pyruvate, β-mercaptoethanol, 50 units/ml penicillin, and 50 mg/ml streptomycin) at 37 °C in a 5% CO_2_ incubator.

### Proliferation assay of primary MEF cells

Primary MEF cells of indicated genotypes were seeded with the same number (1.5 × 10^5^) in a 6-well dish. Cells were passaged every three days and cell numbers were counted. The same number of cells (1.5 × 10^5^) were seeded at each passage.

### Immunofluorescence staining of Phospho-Histone 3 in primary MEF cells

The cells were seeded on coverslips, washed with ice-cold PBS, and fixed with ice-cold methanol for 5 min. The samples were blocked with 5% bovine serum albumin (BSA) in PBST (PBS with 0.1% Tween 20) for 30 min at room temperature, prior to incubation with rabbit anti-pH3-S10 (1:500, #A301–844A, Bethyl) at 4 °C overnight. Next, cells were incubated with the secondary antibody anti-rabbit IgG’ fragment-Cy3 (1:200, C2306, Sigma-Aldrich) for 2 hr at room temperature. The DNA was counterstained with DAPI (40,6-diamidino-2-phenylindole).

### Analysis of γH2AX by immunofluorescence

MEFs were seeded on gelatin-coated coverslips and γ-irradiated with 2 Gy in Gammacell GC40 24 h after plating. At the indicated timepoints, cells were washed with PBS and fixed in PBS containing 4% PFA (Sigma-Aldrich). The blocking was performed using PBS containing 1% BSA (Sigma Aldrich), 0.4% Triton X-100 and 5% normal donkey serum for 2 hr at room temperature. The primary antibody anti-γH2AX (1:200, #05–636, Millipore) was applied overnight at 4 °C. Secondary antibody containing 0.2 µg/ml DAPI (Sigma Aldrich) incubated at room temperature for 1 hr followed by three times of washing before mounting with ProLong Gold Antifade Mountant (P10144, Thermo Fisher). Images of γH2AX foci were examined using Apotome microscope (Carl Zeiss). Foci were counted inside DAPI mask. 300 cells of each samples were randomly chosen for analysis.

### Western blotting

Immunoblotting was carried out as previously described^12^. The immunoblots on PVDF were blocked with 5% BSA in TBST (TBS with 0.1% Tween 20) and washed in TBST. The antibodies used for immunoblotting are as bellow: anti-MCPH1 (1:2000, #4120, Cell Signaling), anti-γH2AX (1:2000, #05–636, Millipore) and anti-β-actin (1:5000, #A5441, Sigma).

### Statistical analysis

The sampling distribution was tested using chi-squared test. Depending on the normality and variance equality, two-tailed Student’s t-test was used. The specific statistical analysis is described in the respective figure legend.

## Results

### Generation of *Mcph1*-ΔBR1 mice

**Fig. 1:**
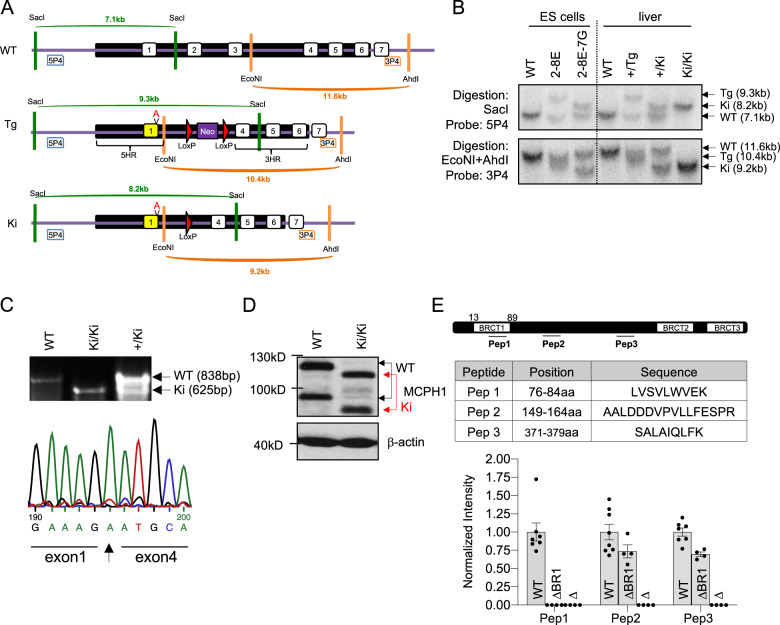
Generation of *Mcph1*-dBR1 knock-in mice. **A** The target strategy to disrupt the N-terminal BRCT domain by deleting exons 2 and 3 in ES cells. After homologous recombination, the targeted allele contains 5′ arm including exon 1 and 3′ arm containing sequence from exon 4 to exon 6. In between, a neomycin cassette (neo, purple box) flanked by two loxP sites (red triangle). One nucleobase adenine (A) (“V”) was inserted at the end of exon 1 (yellow coloured) to keep the truncated protein in frame. Two probes for the Southern blot analysis are indicated, 5P4 (blue box) and 3P4 (orange box). The former detects a fragment of 7.1 kb in the wildtype (WT) allele, 9.3 kb in the targeted (Tg) allele, and 8.2 kb in the knock-in (Ki) allele after genomic DNA digested with SacI. 3P4 detects a fragment of 11.6 kb in WT allele, 10.4 kb in Tg allele, and 9.2 kb in Ki allele after genomic DNA digested with EcoNI and AhdI. The black box represents targeting region; the white box represents exons. **B** Southern blot analysis of targeted ES cells and mice. The genomic DNA of WT, targeted ES clones 2–8E ( + /Tg), and + /Ki ES clones 2–8E-7G, which was obtained after pMC-Cre transfection, were digested by SacI, or EcoNI and AhdI, followed by hybridisation which probes 5P4 (upper panel) and 3P4 (lower panel), respectively. Southern blot analysis of the liver of + /Tg, +/Ki and Ki/Ki mice is also shown to confirm the mutant alleles in these mutant mice. **C** Upper panel: *Mcph1* cDNA was amplified using primer Ex1-F located in exon 1 and Ex8-R located in exon 8 to produce an 838 bp fragment in WT cDNA and 625 bp fragment in Ki cDNA. Lower panel: Sequencing results of *Mcph1*^Ki/Ki^ cDNA containing insA before exon 4. **D** Protein lysates were extracted from WT and *Mcph1*^Ki/Ki^ brains at postnatal day 1 (P1). The MCPH1 protein was examined by Western blotting using an anti-MCPH1 antibody. β-actin was used as a loading control. (E) Position and sequence of the peptides representing the MCPH1 protein used for targeted MS analysis. Pep1 spans the area of BRCT1 domain. Pep2 and Pep3 span the middle domain. Lower panel shows MCPH1 peptide expression profile of the E13.5 neocortex of control (*n* = 7), *Mcph1*-ΔBR1 (*n* = 4) and *Mcph1*-Δ mice (*n* = 4). Note the absence of MCPH1 Pep1, but remained Pep2 and Pep3 peptides in *Mcph1*-ΔBR1 neocortex and complete lacking MCPH1 peptides in *Mcph1*-Δ neocortex.

The N-terminal BRCT domain of MCPH1 (designated as BR1) is mainly encoded by exon 2 and exon 3 (Figure S1A). To delete this domain in a mouse model, the targeting vector was designed with 5′ homologous arm (5HA) containing exon 1 and 3HA containing the entire sequence from exon 4 to exon 6. A neomycin cassette was flanked by two loxP sites, which could be deleted after Cre-mediated recombination (Fig. 1A). In order to avoid the frameshift from exon 1 to exon 4 after deletion of exons 2–3, one nucleobase adenine (A, insA) was inserted at the end of exon 1, prompting an exchange of the sequence GAA (Aspartic acid) with GAT (Glutamic acid) (Fig. 1A). The targeting vector was transfected into embryonic stem cells (ES, E14.1) by electroporation and the targeted events confirmed by Southern blotting. The 5′ external probe 5P4 detected a fragment of 7.1Kb in wildtype (WT) allele, 9.3Kb of targeted (Tg) allele and 8.1Kb in case of knock-in (Ki) allele after SacI digestion of genomic DNA (Fig. 1b, S1B). The 3′ external probe 3P4 detected a fragment of 7.1Kb of WT allele, 9.3Kb of Tg allele and 8.1Kb of Ki allele after EcoNI/AhdI double digestion (Fig. 1b, S1B). Internal probes were used to control random integration of the targeting vectors (Figure S1B). Upon blastocyst injection of the targeted ES clone 2–8E, the *Mcph1* Tg allele successfully went through the mouse germline, which was confirmed by Southern blotting (Fig. 1B) and by PCR genotyping (Figure S1C). After crossing heterozygous *Mcph1*^*+/*Tg^ mice with Stra8-Cre mice that deleted the neomycin cassette, we obtained heterozygous *Mcph1*^+/Ki^ mice. Intercrosses of heterozygous *Mcph1*^+/Ki^ mice produced homozygous *Mcph1*^Ki/Ki^ mice (see below).

To verify whether the mutation is correctly introduced into the germline, we performed cDNA sequencing using primer pair Ex1-Fwd located in exon 1 and Ex8-R located in exon 8 and detected designated insA at the end of exon 1 (Fig. 1C). Western blot analysis revealed a truncated MCPH1 protein from *Mcph1*^Ki/Ki^ brain extracts, due to a loss of 70 aa encoded by exons 2 and 3 (Fig. 1D). To further determine the nature of the truncated MCPH1 protein, we employed targeted mass spectrometry based on parallel reaction monitoring (PRM-MS) using synthetic, isotopically labelled standard peptides covering different regions of MCPH1. PRM-MS was performed on E13.5 (embryonic day 13.5) neocortex of *Mcph1*^Ki/Ki^ mutant mice and confirmed the deletion of the N’-BRCT domain (lacking Pep1) and the presence of the rest of the MCPH1 protein at least from 149aa peptide (determined by Pep2 and Pep3) (Fig. 1E). Of note, the expression level of *Mcph1*^Ki/Ki^ mutant protein is lower, approximately 70% of wildtype (full length) MCPH1, whereas, the *Mcph1*-Δ cortex contained no MCPH1 protein (Fig. 1E). Overall, the *Mcph1*-Ki allele expresses expected mutant MCPH1 mRNA and protein lacking the N-terminal BRCT domain. Thus, the homozygous *Mcph1*^Ki/Ki^ mutation was designated *Mcph1*-ΔBR1.

### *Mcph1*-ΔBR1 mice display postnatal growth retardation and primary microcephaly

Although *Mcph1*-ΔBR1 mice were viable, the amount of newborn pups of the *Mcph1*-ΔBR1 genotype from heterozygous (*Mcph1*^+/Ki^) intercrosses was lower than expected according to the Mendelian rule, similar to *Mcph1*-Δ mouse production (Figure S1A). *Mcph1*-ΔBR1 mice had smaller body weight in both female and male compared to wildtype (WT) mice, readily at P5 (postnatal day 5) after birth (Figure S1B, C), indicating that N’-BRCT deletion results in growth retardation, as in MCPH1 complete knockout mice^22,23^. Given the lack of phenotypes among heterozygous + /Ki or + /Δ and WT mice (data not shown, see below), we used heterozygous as well as WT mice as controls in the following experiments.

**Fig. 2:**
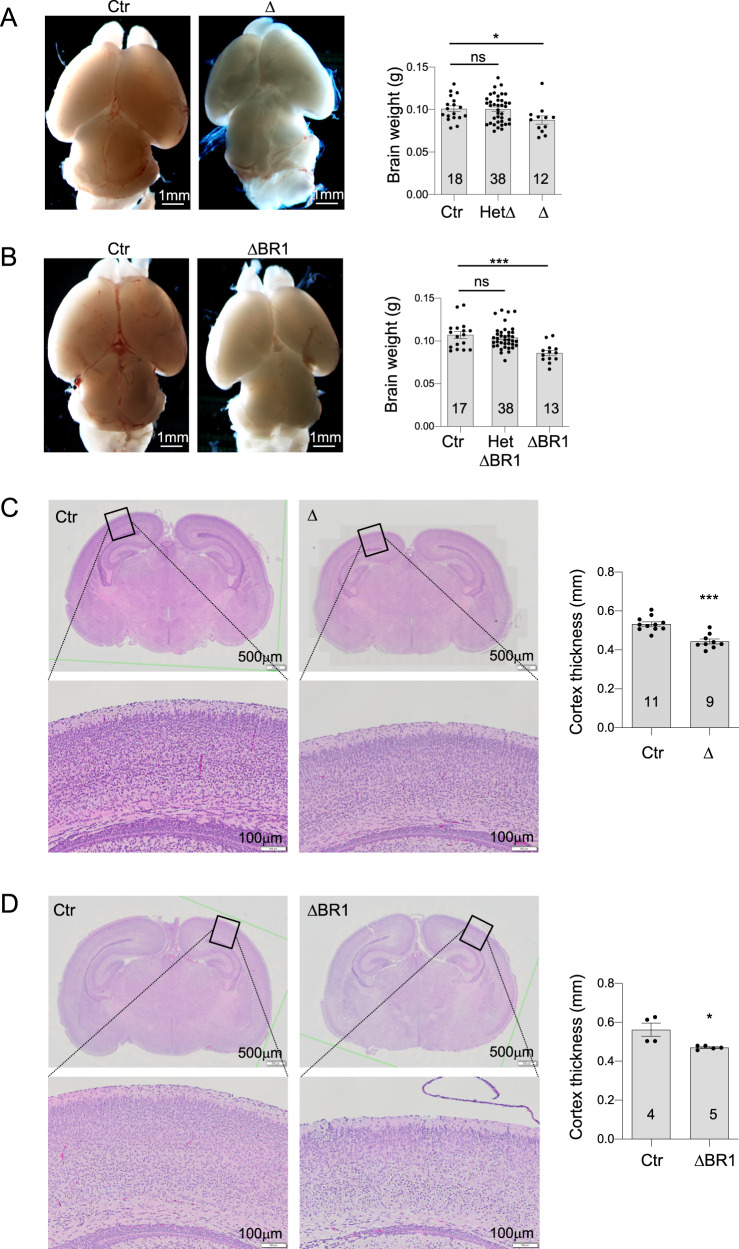
Mcph1-ΔBR1 mice develop microcephaly. **A** Left panel: Images of the brain of control and *Mcph1*-Δ (Δ) mice at postnatal day P1. Right panel: Quantification of brain weight of wildtype control (Ctr), heterozygous *Mcph1*-Δ (HetΔ) and homozygous *Mcph1*-Δ (Δ) mice. **B** Left panel: Images of the brain of control and *Mcph1*-ΔBR1 (ΔBR1) mice at postnatal day P1. Right panel: Quantification of brain weight of WT, heterozygous + /Ki (HetΔBR1) and homozygous Ki/Ki (ΔBR1) mice. **C** Hematoxylin and Eosin (H&E) staining of coronal sections of control and *Mcph1*-Δ brains at P1. Left panel: Overview of coronal brain sections and an enlarged view of the forebrain cortex from the rectangular areas of the upper panel are shown below. Right panel: Quantification of the thickness of the cerebral cortex is shown. **D** H&E staining of coronal sections of control and *Mcph1*-ΔBR1 brains at P1. Left panel: Overview of coronal brain sections and an enlarged view of the forebrain cortex from the rectangular areas of the upper panel are shown below. Right panel: Quantification of the thickness of the cerebral cortex is shown. The number of mice used is indicated within the graph bars. Bars represent the S.D. Statistical analysis was performed by Student’s t-test. *p < 0.05; ***p < 0.001; ns, not significant.

**Fig. 3:**
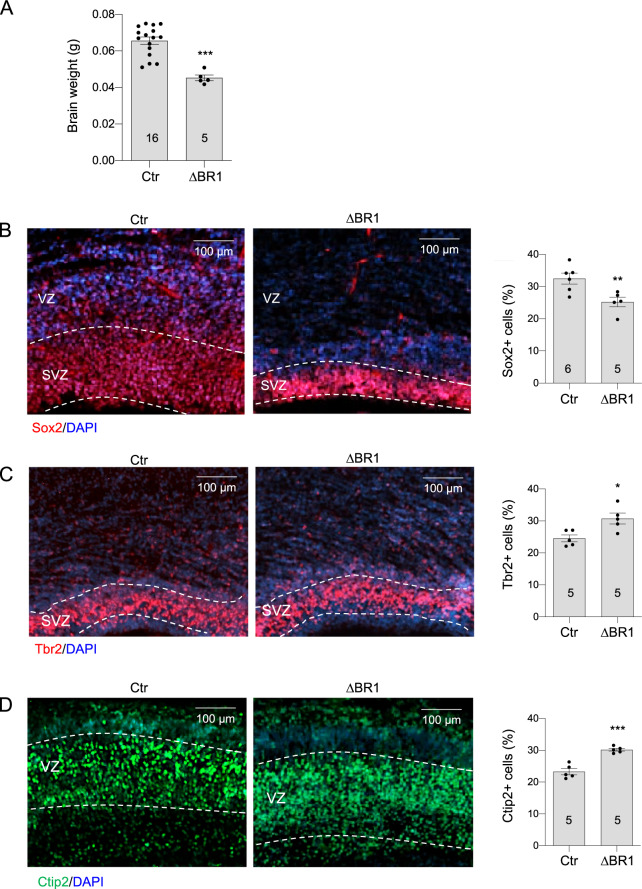
Histological analysis of the neocortex at E17.5. **A** Brain weight of control (Ctr) and *Mcph1*-ΔBR1 (ΔBR1) mice at E17.5. **B** Immunostaining of Sox2 on coronal sections of control and *Mcph1*-ΔBR1 brain at E17.5. Right panel: Quantification of percentage of Sox2 positive cells in control and *Mcph1*-ΔBR1 mice. **C** Immunostaining of Tbr2 in control and *Mcph1*-ΔBR1 mice at E17.5. Right panel: Quantification of percentage of Ctip and Tbr2 positive cells in control and *Mcph1*-ΔBR1 mice. **D** Immunostaining of Ctip2 in control and *Mcph1*-ΔBR1 mice at E17.5. Right panel: Quantification of percentage of Ctip and Tbr2 positive cells in control and *Mcph1*-ΔBR1 mice. VZ: ventricular zone. SVZ: subventicular zone. IZ: intermediate zone. The number of mice used is indicated within the graph bars. Bars represent the S.D. Statistical analysis was performed by Student’s t-test. *p < 0.05; **p < 0.01; ***p < 0.001.

Similar to *Mcph1*-Δ mice^23,26^, *Mcph1*-ΔBR1 mice displayed smaller brain size and their brain weight was significantly smaller compared to control mice at P1 (Fig. 2A, B). Hematoxylin and Eosin (H&E) staining revealed another similarity in that *Mcph1*-ΔBR1 mice had a thinner neocortex compared to controls (Fig. 2C, D). The brain weight of *Mcph1*-ΔBR1 mice at E17.5 was already smaller (Fig. 3A). These findings demonstrate a primary microcephaly in *Mcph1*-ΔBR1 mice. We examined neuroprogenitors and newborn neurons at E17.5, to investigate the effect of the deletion of the N-terminal BRCT domain in brain development. By staining the cerebral cortex with Sox2 (a marker for neuroprogenitors in layer I), Tbr2 (a marker for intermediate neuroprogenitors in layer II) and Ctip2 (a marker for neurons in layer VI), we found that the percentage of Sox2+ cells was significantly lower in the *Mcph1*-ΔBR1 cortex (Fig. 3B); whereas, the percentage of Tbr1+ and Ctip2+ cells was significantly higher in the *Mcph1*-ΔBR1 neocortex compared to controls (Fig. 3C, D), reminiscent of the *Mcph1*-Δ brain^23^. Thus, the N’-BRCT is as important as the entire MCPH1 protein in maintaining the neuroprogenitor pool and preventing primary microcephaly.

### PCC and DDR defects of *Mcph1*-ΔBR1 MEF cells

**Fig. 4:**
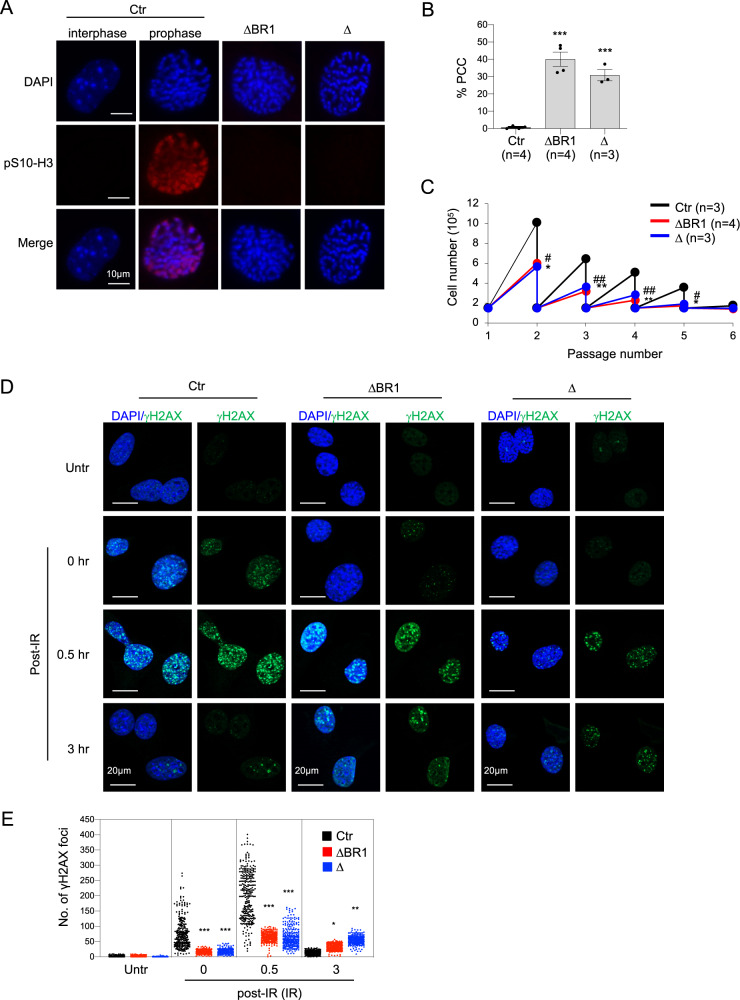
Cellular defects of Mcph1-ΔBR1 MEFs. **A** PCC is observed in *Mcph1*-Δ (Δ) and *Mcph1*-ΔBR1 (ΔBR1) primary MEF cells. Representative images of normal interphase nuclei and chromosome condensation (prophase) together with pS10-H3 staining in control primary MEF cells. Note highly condensed chromosomes (visualised by DAPI staining) lacking pS10-H3 staining in *Mcph1*-Δ and *Mcph1*-ΔBR1 primary MEF cells after DAPI staining. The number of cell lines of each genotype (n) used for measurement is indicated. **B** Quantification of PCC-containing cells in primary MEF cells. More than 1000 cells of each genotype were counted for each group. The number of cell lines of the indicated genotype (n) are indicated. **C** Proliferation defects of *Mcph1*-Δ and *Mcph1*-ΔBR1 primary MEF cells. The proliferation rate was measured by seeding the same amount (1.5 × 10^5^) of primary MEF cells of indicated genotypes. The cells were passaged every three days and the cell numbers determined before passaging. The experiment was repeated three times. **D** Representative images of γH2AX staining on MEF cells of the indicated genotype, either untreated (Untr) or treated with 2 Gy ionising radiation (IR) at the indicated recovery time. **E** Quantification of the number of γH2AX foci in cells untreated (Untr) or treated with 2 Gy IR and analysed at 0 hr, 0.5 hr or 3 hr post-IR. 300 cells of each sample were scored. The experiment was repeated three times. Bars represent the S.D. Statistical analysis was performed by Student’s t-test. *^, #^*p* < 0.05; **^, ##^*p* < 0.01; ***p < 0.001.

PCC is a prominent cellular characteristic of both human microcephaly patients and the *Mcph1*-Δ mouse model^9,11,23^. To study the cellular effect of N’-BRCT deletion, we isolated primary MEF cells from E13.5 embryos of WT, *Mcph1*-ΔBR1 and *Mcph1*-Δ genotypes. The cells were stained with mitotic marker phospho-histone H3 (pS10-H3) and counterstained with DAPI, which allows visualisation of chromosome condensation. We scored PCC cells that display chromosome condensation by DAPI staining, but negative for pS10-H3 signals. The incidence of PCC cells was significantly higher in *Mcph1*-ΔBR1 MEFs, similar to *Mcph1*-Δ, compared to controls (Fig. 4A, B). Next we examined the proliferation capacity of *Mcph1*-ΔBR1 primary MEF cells by a 3T3 protocol. Initially, 1.5 × 10^5^ cells of the desired genotypes were seeded, cells counted every three days and reseeded at the same number. As shown in Fig. 4C, *Mcph1*-ΔBR1 MEFs proliferated at a similar rate to *Mcph1*-Δ MEFs, but much slower than control cells, with a loss of proliferation capacity two passages earlier than controls. Thus, MCPH1 mutated cells have impaired proliferation and prematurely enter cellular senescence.

MCPH1 is involved in the DDR^24,25,35^ and *Mcph1*-Δ neuroprogenitors are hypersensitive to ionising radiation (IR)^26^. We next studied the DDR of *Mcph1*-ΔBR1 by treating MEF cells with 2 Gy IR and stained with antibodies against γH2AX, a DNA damage marker, at 0 hr, 0.5 hr or 3 hr after irradiation. The *Mcph1*-ΔBR1 cells and *Mcph1*-Δ cells displayed a significantly lower number of γH2AX foci at 0 hr and 0.5 hr post-IR, but maintained a higher γH2AX level at 3 hr post-IR, when compared to controls (Fig. 4D, E). Western blotting confirmed the kinetics of γH2AX accumulation after IR (Figure S3A). These findings together indicate an impaired DDR signalling and compromised DNA repair in both *Mcph1*-ΔBR1 cells and *Mcph1*-Δ cells.

### *Mcph1*-ΔBR1 mice are infertile because of gonad developmental defects

**Fig. 5:**
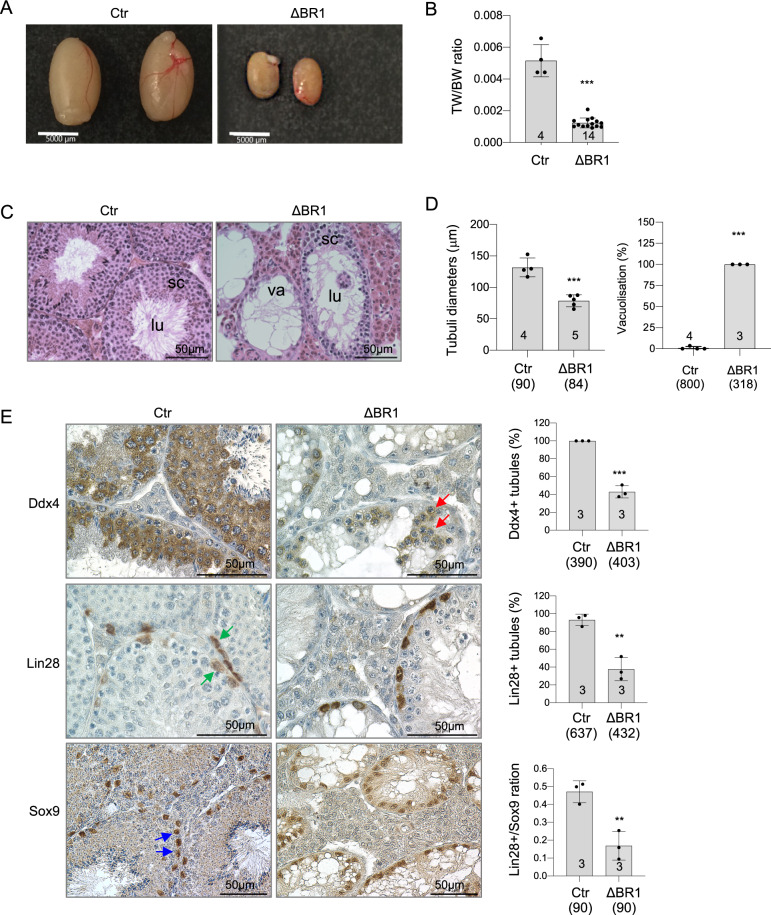
Mcph1-ΔBR1 mice show developmental block in reproductive organs. **A** Macroscopic view of testes of control and *Mcph1*-ΔBR1 mice. Testis size is strongly reduced in *Mcph1*-ΔBR1 mice compared to controls (Ctr). **B** Ratio of paired testis weight to body weight (TW/BW) is significantly lower in *Mcph1*-ΔBR1 mice compared to controls. The number of mice analysed is indicated within the bar. **C** Representative pictures of H&E stained testis sections demonstrate a strong degeneration of the testicular germinal epithelium in *Mcph1*-ΔBR1 mice with lack of post-meiotic cells. Note that mutant testis tubules contain either few or lack of spermatocytes (sc) and vacuolised lumens (lu). **D** Quantitative histological analyses of H&E stained testis sections reveal significantly smaller diameter of seminiferous tubules and a strong vacuolisation of the germinal epithelium in *Mcph1*-ΔBR1 mice. Each circle represents one Ctr mouse and each diamond one *Mcph1*-ΔBR1 mouse. Per mouse, 30 circular tubular cross-sections were analysed to determine the diameter, and a minimum of 70 tubular cross-sections per mouse have been evaluated for presence or absence of vacuoles (va). The number of mice analysed is indicated within the bar and the number of analysed (circular) tubular cross-sections is indicated under the genotype in parentheses. **E** Representative pictures of immunostaining of testis sections by the indicated antibodies. DEAD box helicase 4 (Ddx4) germ cell marker staining detects residual germ cells (red arrows) in mutant testes. Lin-28 homologue A (Lin28) is expressed by spermatogonial stem cells and progenitors (green arrows). SRY-box 9 (Sox9) marker protein is expressed normally in Sertoli cells (blue arrows) of mutants and controls. Intense brown colour indicates positive marker staining, blue colour depicts hematoxylin counterstaining. Quantification of marker proteins is shown on the right panel of respective immunostaining. The number of mice analysed is indicated within the bar. The number of tubuli analysed is indicated under the genotype in parentheses. All experiments have been performed on testicular cross-sections of adult mice (9.5–12 months old). Bars represent Means ± SEM of a minimum of 3 determinations. Statistical analysis was performed by Student’s t-test. **p < 0.01, ***p < 0.001.

As with *Mcph1*-Δ mice^23^, we could not obtain any offspring from intercrosses or backcrosses of *Mcph1*-ΔBR1 mice, suggesting that the N’-BRCT domain is required for mouse fertility. We then investigated whether testicular germ cells, as well as somatic cells of mutant animals are affected by the loss of the N-terminal BRCT. Histological analysis of 9.5–12 month-old males revealed *Mcph1*-ΔBR1 mutant male mice to have smaller testes, similar to *Mcph1*-Δ mice, in stark contrast to control mice (Fig. 5A, B). No mature spermatozoa could be found in the lumen of testicular tubules of *Mcph1*-Δ and *Mcph1*-ΔBR1 mice (Fig. 5C, S4A, B). H&E staining illustrated significantly smaller diameter of seminiferous tubules (average diameter of controls: 131,7 µm versus *Mcph1*-ΔBR1: 78,7 µm) and a strong vacuolisation of the germinal epithelium in *Mcph1*-ΔBR1 mice (Fig. 5C, D, S4A, B). In order to study whether germ cells are still present in seminiferous tubules of adult *Mcph1*-ΔBR1 mice, immunohistochemical analyses were performed on testis sections. DEAD box helicase 4 (Ddx4) staining revealed a strong reduction of germ cell-containing mutant tubules (~43% Ddx4+ tubules and 57% Sertoli cell-only tubules) (Fig. 5E). Interestingly, Lin28+ spermatogonial stem cells (SSCs) were still present in *Mcph1*-ΔBR1 testes (approx. 38% of mutant tubules with Lin28+ SSCs versus approx. 93% control tubules with Lin28+ SSCs); although the number of Lin28+ cells in seminiferous tubules of mutant mice was significantly lower and the ratio of Lin28+ SSCs to SRY-box 9 positive (Sox9+) Sertoli cells is 2.8-fold lower in *Mcph1*-ΔBR1 mice compared to controls (Fig. 5E). A strongly reduced number of Ddx4-positive seminiferous tubules per testicular cross-section accompanies an increase in Sertoli cell-only tubules in mutant mice (Sox9 staining). Quantification of Sox9 and Lin28 markers shows a significant reduction of the number of Ddx4+ and Lin28+ SSCs and progenitors in testes of *Mcph1*-∆BR1 mice compared to controls.

Of note, the presence of Sertoli cells that express the androgen receptor (Ar) was normal in mutant mice (Figure S4C) and these cells still stained positive for connexin 43 (Cx43), a gap junction protein critical to formation of the blood-testis-barrier (Figure S4D). However, the blood-testis barrier in *Mcph1*-ΔBR1 testes could not be established compared to controls, due to germ cell degeneration (Figure S4D). Moreover, we observed a normal pattern of testosterone-producing Leydig cells positive for 3β-hydroxysteroid dehydrogenase (3β-Hsd), an enzyme involved in the steroid hormone synthesis (Figure S4E). The seemingly higher number of 3β-Hsd+ cells might be a compensatory response to the degeneration or disruption of testicular seminiferous epithelium. These results indicate that both Sertoli and Leydig cells are functional and unaffected by the deletion. All in all, these results demonstrate an impaired meiotic progression and a strongly degenerated testicular seminiferous epithelium in mice lacking the N-terminal BRCT, while testicular niche cells are not affected.

**Fig. 6:**
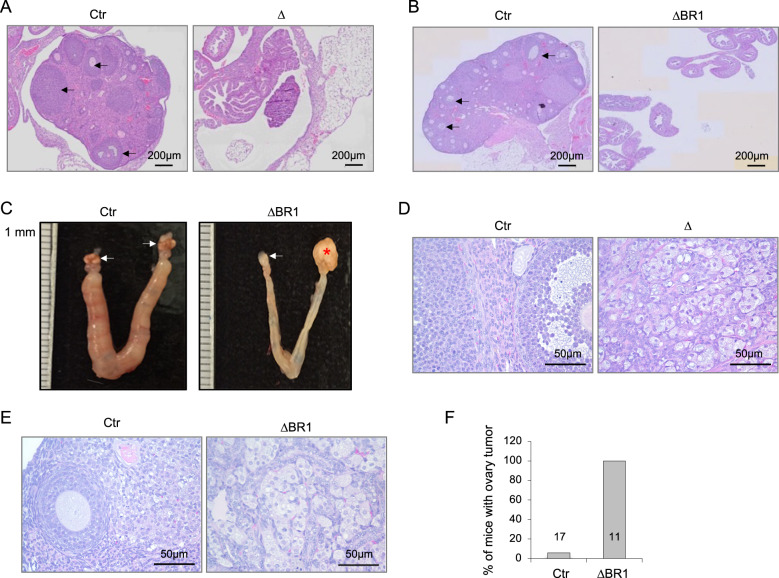
Blockage of ovary development and ovary tumorigenesis in Mcph1 mutant mice. **A** H&E staining of ovary sections from 1-month-old control (Ctr) and *Mcph1*-Δ (Δ) female mice. Arrows point to follicles. **B** H&E staining of ovary sections from 2-month-old control and *Mcph1*-ΔBR1 (ΔBR1) female mice. Arrows point to follicles. **C** Macroscopic images of ovarian tumours of *Mcph1*-ΔBR1 female mice. Arrows point to ovaries. Asterisk* marks ovary tumour mass. **D** H&E staining of ovary sections from 12-month-old control and *Mcph1*-Δ mice. Histopathology revealed benign tubular adenoma. **E** H&E staining of ovary sections from 12-month-old controls and *Mcph1*-ΔBR1 mice. **F** The percentage of tumour incidence in control and *Mcph1*-ΔBR1 female mice at 12–18 months of age. The number of mice analysed is indicated within the bar.

When examining female infertility, we failed to observe the ovary macroscopically in 2-month-old females of both *Mcph1*-ΔBR1 and *Mcph1*-Δ mice (Fig. 6A, B). So as to study the reason for infertility, histological analyses were performed on H&E stained ovary sections of mutant and control mice. Ovaries of *Mcph1*-ΔBR1 and *Mcph1*-Δ young female mice were significantly smaller and lacking obvious follicles and oocytes (Fig. 6A, B). Strikingly, we noticed the occurrence of ovarian tumours in aged *Mcph1*-ΔBR1 mice (Fig. 6C–E) with a high penetrance (Fig. 6F), as observed in *Mcph1*-Δ females^27^. Of note, no testicular tumours were observed. These findings indicate that the N-terminal BRCT domain is required for gonad development and prevents ovary tumorigenesis in old female mice.

## Discussions

MCPH1 consists of one N-terminal BRCT domain and two C-terminal BRCT domains. Most, if not all, MCPH1 mutations in human patients are found in the N-terminal part of the protein^13,27^, suggesting the importance of this part of MCPH1 in brain development. Here, we show that indeed the N-terminal BRCT domain is a decisive element in preventing microcephaly and also PCC, both prominent hallmarks of MCPH1 patients. Strikingly, *Mcph1*-ΔBR1 mice completely recapitulate the brain developmental deficits of *Mcph1* null mice (*Mcph1*-ko and *Mcph1*-Δ), including primary microcephaly, thinner neocortex with reduced neuroprogenitor pools and premature neuronal differentiation. Similar to *Mcph1*-ko^22^ and *Mcph1*-Δ^23^ mice, *Mcph1*-ΔBR1 mice were infertile, with males showing testis atrophy without mature sperms due to block of meiosis and females lacking overt ovary structures. Intriguingly, both *Mcph1*-ΔBR1 and *Mcph1*-Δ female mice developed high incidence of ovary tumours in a period of 18 months (^27^ and current study). Akin to complete deletion of MCPH1^23,26^ and MCPH1 patient cells^24,25^, *Mcph1*-ΔBR1 cells show prominent PCC phenotype and defects in MEF cell proliferation. Interestingly, different from a previous study showing that the N’-BRCT is dispensable for γH2AX focus formation when assaying ectopically expressed MCPH1 truncation mutants in cells^35^, our cellular analyses demonstrate that the N-terminal BRCT domain is indeed required for the efficient initiation of the DDR and DNA repair. In conclusion, the N-terminal BRCT domain has a functional importance equivalent to full length MCPH1 in vivo.

In humans, four isoforms of MCPH1 are expressed in cells, including MCPH1-FL, MCPH1∆e1–3, ∆e9–14, and ∆e8^36^. MCPH1∆e1–3 lacking N-terminal BRCT domain may not be physiologically expressed, while MCPH1∆e9–14 that miss two C-terminal BRCT domains are highly expressed at the foetal stage of the brain, heart, and thymus, similar to MCPH1-FL^36^ – suggesting the isoform lacking the C-terminal BRCT domains remains functional. In this regard, it is notable that mice lacking the C-terminal BRCT domain (*Mcph1*^gt/gt^ mice) did not exhibit microcephaly, although mutant cells showed PCC^29^. Altogether, these findings suggest that the entire protein is required for repressing PCC and the N-terminal BRCT is essential for brain and gonad development.

It is interesting to note that deletion of the N-terminal BRCT domain faithfully recapitulates the microcephaly and other phenotypes in mice lacking the entire MCPH1 protein. We showed previously that a complete depletion of MCPH1 caused premature mitotic entry with hypo-phosphorylation of Cdk1 via Chk1-dependent and βTrCP2-mediated Cdc25 regulation^12,23^. These molecular pathways are responsible for the neuroprogenitor division mode and maintenance of neuroprogenitor pools, thereby preventing microcephaly^12,23^. Yet, both Chk1 and βTrCP2 bind the middle region of MCPH1, but not N-terminal BRCT domain^12,17^. Of note there is a recently published paper reporting that two novel compound heterozygous missense variants (c.982 G > A and c.1273 T > A) in the exon 8 of MCPH1 gene caused primary microcephaly in a Saudi family; indicating an important role of middle regions of MCPH1 for brain development in addition to the N-terminal BRCT domain^37^. Because *Mcph1*-ΔBR1 mutation maintained the intact middle region required for the interaction between MCPH1 and βTrCP2, these data suggest that the N-terminal BRCT domain has a function to regulate neurogenesis likely beyond the MCPH1-βTrCP2-Cdc25 axis. But we cannot disregard the possibility that a physical cross-talk between the N’-BRCT and the middle region is necessary in a conformation-dependent manner.

Both our *Mcph1*-Δ and *Mcph1*-ΔBR1 models and another *Mcph1*-ko model^22^, all exhibited infertility with atrophy in reproductive organs, i.e. testes and ovaries^12,22^. Currently, no case report exists on human primary microcephaly associated with infertility, or testicular or ovarian atrophy. This raises an interesting question as to whether positive selection of MCPH1 during primate and human lineage evolution links brain size expansion with the germline fitness. Interestingly, *Mcph1*-Δ and *Mcph1*-ΔBR1 develop a very high incidence of tumours, yet only in ovaries, in great contrast to 17.1% *Mcph1*-ko mice, which developed malignant tumours originated from lymphomas and granulosa ovary tumours^22^. Different tumour spectrum and penetrance between these mice are unclear, but are likely attributable to genetic background and mutation introduced in these mice. Nonetheless, a high tumour incidence of these *Mcph1*-Δ and *Mcph1*-ΔBR1 mice supports tumour repressor function of MCPH1, corresponding to previous studies showing that MCPH1 is down-regulated or mutated in human cancer patients, including those suffering ovarian cancer^24,38,39^.

The identical phenotypes –small brain size and neuroprogenitor defects, PCC, DDR defects, the developmental block of gonads, as well as tumorigenesis – among N’-BRCT-deleted (*Mcph1*-ΔBR1) and MCPH1 complete knockout (*Mcph1*-ko, *Mcph1*-Δ) mice, strongly suggest an essential function of the N-terminal BRCT domain of the MCPH1 protein in brain size determination and other biological processes. MCPH1 has no enzymatic activity and functions most likely as a scaffold protein participating in various cellular activities. Therefore, the interaction partners of the N-terminal BRCT domain seem to coordinate the neuroprogenitor fate and pools in brain size determination, gonad development and tumorigenesis.

## Supplementary information

Suppl Figure legends

Suppl Figure 1

Suppl Figure 2

Suppl Figure 3

Suppl Figure 4
