# Supplementary material for: The N-terminal BRCT domain determines MCPH1 function in brain development and fertility
Source: Cell Death Dis. 2021 Feb 1;12(2):143. doi: 10.1038/s41419-021-03406-3 (PMC7862653; doi:10.1038/s41419-021-03406-3)
Supplement: Supplementary file 1 — Suppl Figure legends [file 41419_2021_3406_MOESM1_ESM.docx]

## **The N-terminal BRCT domain determines MCPH1 function in brain development and fertility**

Xiaoqian Liu, et al.

**Supplementary Figures**

**Figure S1. Characterisation of *Mcph1* knock-in ES cells and mice.**

(A) Schematic presentation of mouse MCPH1 protein and gene locus. Mouse MCPH1 protein contains three BRCT domains: BRCT1 in the N-terminus and BCRT2-3 in the C-terminus. The *Mcph1* gene contains 14 exons and BRCT1 (13-89aa) domain is encoded by the entire exons 2, 3 and partial exons 1 and 4. White box represents exons.

(B) Southern blot analysis of targeted ES cells. The genomic DNA of ES clones after gene targeting was digested by SacI (left panel), or EcoNI and AhdI (right panel), followed by hybridisation with external probes 5P4 and 3P4 (upper panel) and internal probes 5P3 and pNeo (lower panel), respectively. Arrows point to WT and Tg alleles.

(C) Upper panel: A PCR strategy to identify the WT, Tg, and Ki allele is presented. Four primers are designed: Fwd Ex1 located in exon 1, I1as in intron 1, Rev Neo in neomycin cassette, and Rev Ex4 in exon 4. The primer pair Fwd Ex1 and I1as amplify a 312bp fragment in the WT allele. The primer pair Fwd Ex1 and Rev Neo amplify a 582bp fragment in the Tg allele. The primer pair Fwd Ex1 and Rev Ex4 amplify a 462bp fragment in the Ki allele. Lower panel: PCR results of genome DNA from WT, +/Tg. +/Ki and Ki/Ki mice.

**Figure S2: *Mcph1*-ΔBR1 mice show lower Mendelian ratio and postnatal growth retardation.**

(A) Distribution of offspring from intercross of heterozygous +/Δ (left panel) or +/Ki (right panel) mice. The number of offspring and expected Mendelian ratio (%) are listed in the table.

(B) Body weight of *Mcph1*-Δ female (left panel) and male pups (right panel) at postnatal days P5, P10, P15, and P20. The number of mice in each group of wildtype control (Ctr), heterozygous +/Δ (HetΔ) and homozygous Δ/Δ (Δ) is shown in panel (A) left table panel.

(C) Body weight of *Mcph1*- ΔBR1 female (left panel) and male pups (right panel) at postnatal days P5, P10, P15, and P20. The number of mice in each group of wildtype control (Ctr), heterozygous +/Ki (HetΔBR1) and homozygous Ki/Ki (ΔBR1) is shown in panel (A) right table panel.

Bars represent the SEM. Statistical analysis was performed by Student’s t-test. *, p<0.05; **, p<0.01.

**Figure S3. Western blot analysis of DDR.**

(A) MEF cells were untreated (Untr) or treated with 2 Gy IR and analysed at 0 hr, 0.5 hr or 3 hr post-IR using anti-γH2AX antibody. β-actin was used as a loading control. The experiment was repeated three times and the representative blots are shown.

**Figure S4. MCPH1 mutant mice have atrophic testes but normal somatic cell compartments.**

(A) H&E staining of testis from 3-month-old control (Ctr) and *Mcph1*-Δ (Δ) mice.

(B) H&E staining of testis from 2-month-old control and *Mcph1*-ΔBR1 (ΔBR1) mice.

Note that MCPH1 mutant testis tubules lack spermatocytes (sc) and contain vacuolised (Va) lumens (lu).

(C) Androgen receptor (Ar) expression in Sertoli cells indicate functionally mature somatic cells in testes of control (Ctr) and *Mcph1*-ΔBR1 (ΔBR1) mice. Number of Ar-positive seminiferous tubules per testicular cross-section indicates no difference between control and mutant mice.

(D) Connexin 43 protein (Cx43) is expressed by Sertoli cells of controls and *Mcph1*-ΔBR1 mice. Black arrows indicate an intact blood-testis barrier in controls, which could not be established in mutant mice due to germ cell degeneration.

(E) 3ß-hydroxysteroid dehydrogenase (3β-Hsd) expression in interstitial Leydig cells.

(F) Goat (gIgG) and rabbit IgG (rIgG) staining on control testis sections represents isotype controls for Figure 5E, S4C-E.

Intense brown colour indicates positive marker staining, the blue colour depicts hematoxylin counterstaining. The number of mice analysed is indicated within the bar. The number of tubuli analysed is indicated under the genotype in parentheses. All experiments were performed on testicular cross-sections of adult mice (9.5-12 months old).
