# Supplementary figures and images for: The N-terminal BRCT domain determines MCPH1 function in brain development and fertility
Source: Cell Death Dis. 2021 Feb 1;12(2):143. doi: 10.1038/s41419-021-03406-3 (PMC7862653; doi:10.1038/s41419-021-03406-3)

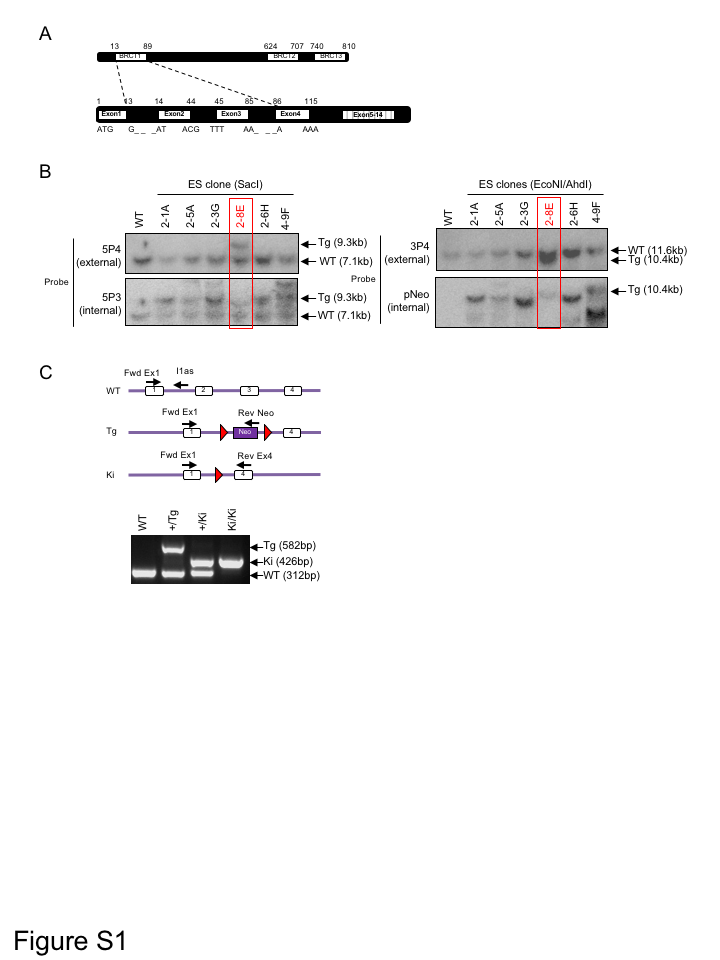

Supplement: Supplementary file 2 — Suppl Figure 1 [file 41419_2021_3406_MOESM2_ESM.tif]

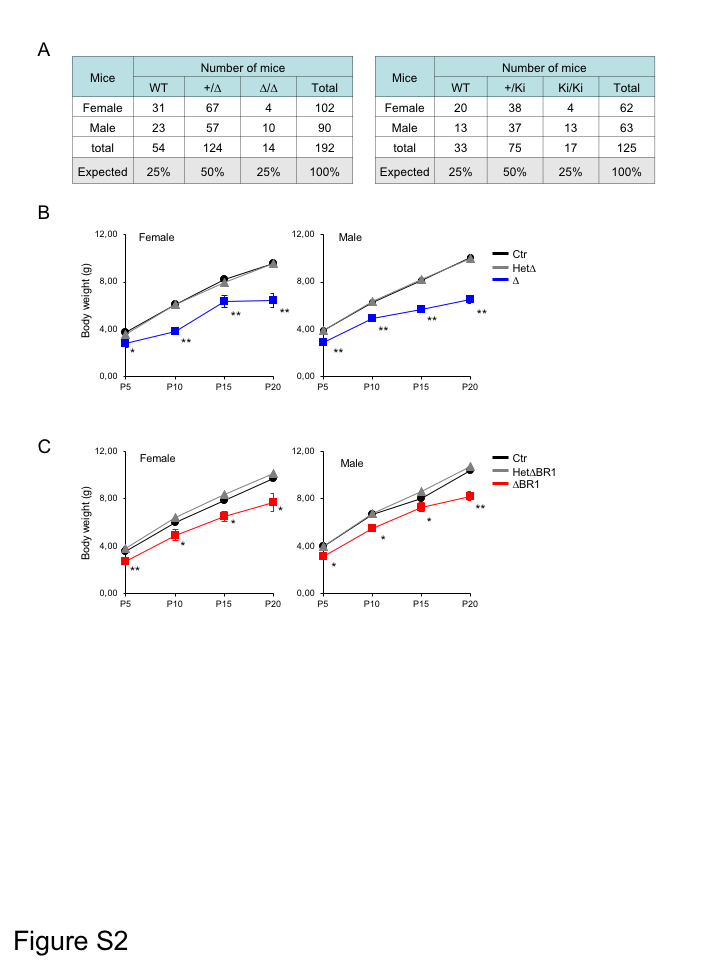

Supplement: Supplementary file 3 — Suppl Figure 2 [file 41419_2021_3406_MOESM3_ESM.tif]

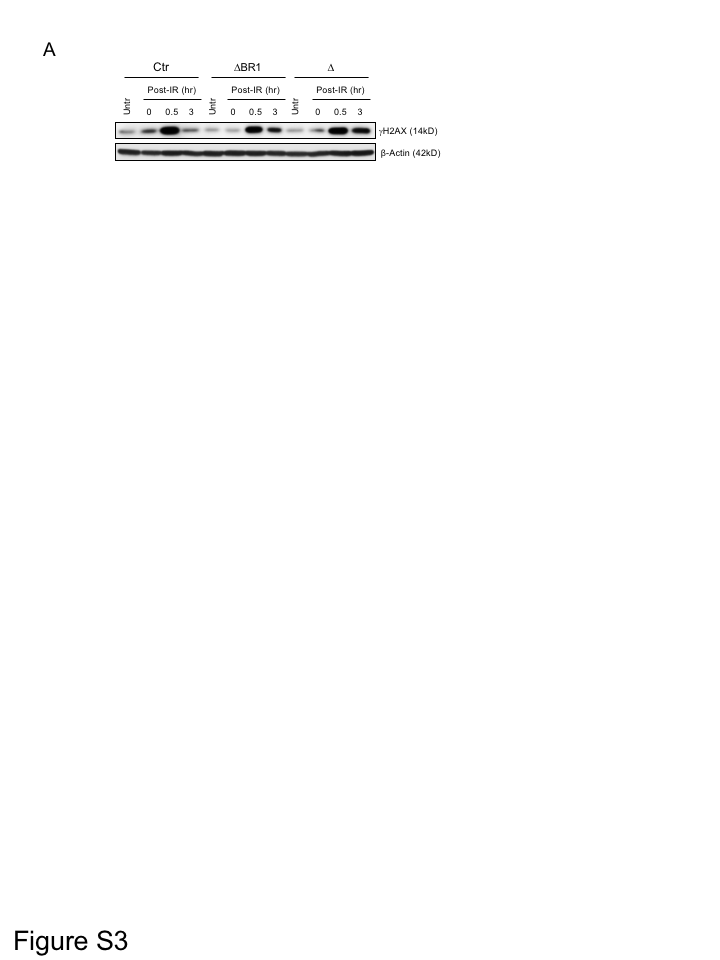

Supplement: Supplementary file 4 — Suppl Figure 3 [file 41419_2021_3406_MOESM4_ESM.tif]

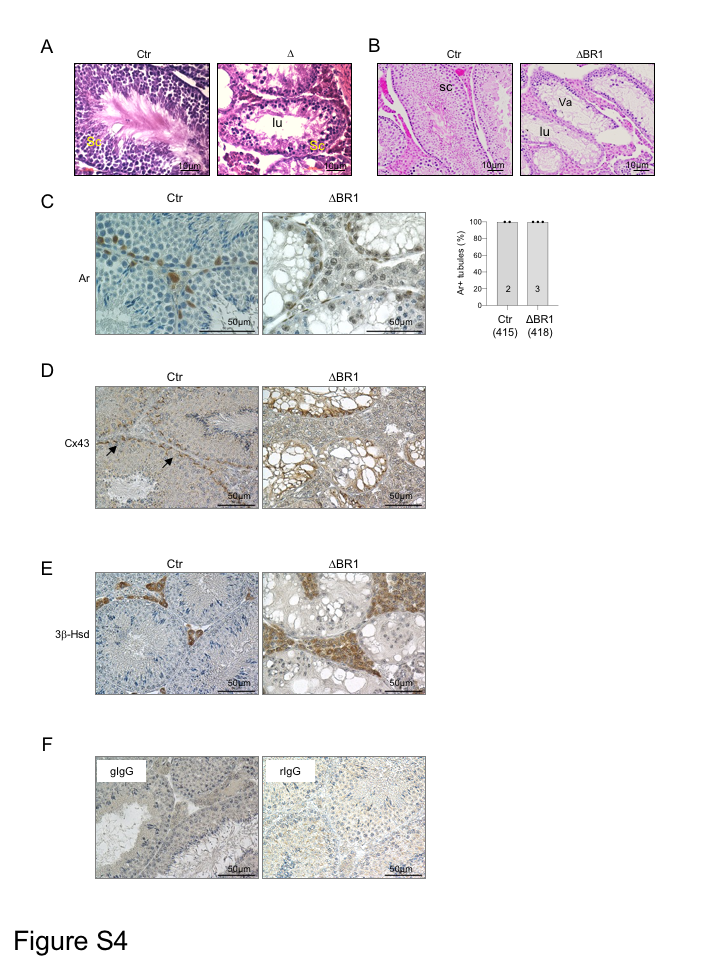

Supplement: Supplementary file 5 — Suppl Figure 4 [file 41419_2021_3406_MOESM5_ESM.tif]
